# Combination of an Axicon Fiber Tip and a Camera Device into a Sensitive Refractive Index Sensor

**DOI:** 10.3390/s19224911

**Published:** 2019-11-11

**Authors:** Yi-Hsin Tai, Po-Cheng Tsai, Ya-Lun Ho, Jean-Jacques Delaunay, Pei-Kuen Wei

**Affiliations:** 1 Department of Mechanical Engineering, School of Engineering, The University of Tokyo, Tokyo 113-8656, Japan; tai.yi-hsin@scale.t.u-tokyo.ac.jp (Y.-H.T.); ho.ya-lun@scale.t.u-tokyo.ac.jp (Y.-L.H.); jean@mech.t.u-tokyo.ac.jp (J.-J.D.); 2 Graduate Institute of Electronics Engineering, National Taiwan University, Taipei 10617, Taiwan; d07943004@ntu.edu.tw; 3 Research Center for Applied Sciences, Academia Sinica, Taipei 11529, Taiwan; 4 Institute of Biophotonics, National Yang-Ming University, Taipei 11221, Taiwan; 5 Institute of Optoelectronic Sciences, National Taiwan Ocean University, Keelung 20224, Taiwan

**Keywords:** axicon optical fiber, refractometer, smartphone, intensity sensitivity

## Abstract

An axicon fiber tip combined with a camera device is developed to sensitively detect refractive indexes in solutions. The transparent axicon tips were made by etching optical fibers through a wet end-etching method at room temperature. When the axicon fiber tip was immersed in various refractive index media, the angular spectrum of the emitted light from the axicon fiber tip was changed. Using a low numerical aperture lens to collect the directly transmitted light, a high intensity sensitivity was achieved when the tip cone angle was about 35 to 40 degrees. We combined the axicon fiber tip with a laser diode and a smartphone into a portable refractometer. The front camera of the smartphone was used to collect the light emitted from the axicon fiber tip. By analyzing the selected area of the captured images, the refractive index can be distinguished for various solutions. The refractive index sensitivity was up to 56,000%/RIU, and the detection limit was 1.79 × 10^−5^ RIU. By measuring the refractive index change via the axicon fiber tip, the concentration of different mediums can be sensitively detected. The detection limits of the measurement for sucrose solutions, saline solutions, and diluted wine were 8.86 × 10^−3^ °Bx, 0.12‰, and 0.35%, respectively.

## 1. Introduction

The refractive index is a standard used for proving the quality or concentration in aqueous solutions. By measuring the refractive index, the change of the concentration or differing quality of the solution can be rapidly detected. For food detection, the sugar or salt content in solutions [1] can be measured by a refractometer. Moreover, the proteinuria concentration in urine [2], water quality [3], or assay of industrial solutions [4] can be rapidly distinguished by measuring refractive index changes. Currently, the popular method for refractive measurement uses the total reflection in an optical prism [5]. According to Snell’s law, the difference in the refractive index between the solution and the prism makes the critical angle of incidence change. By measuring the angle of the total internal reflection or refractive angle, the refractive index of the sample solution can be determined. Compared to a prism-based refractometer, an optical fiber-based refractometer contains the advantages of easy detection, flexibility, and small sample volume. Different types of fiber-based refractometers have been developed, such as tapered fibers [6,7], metal-coated fibers [8,9], and fiber with period structures [10,11,12]. This work introduces a high-sensitivity measurement method by using a transparent axicon optical fiber tip combined with a camera device to detect the refractive index of various liquids. The input light from a laser diode is coupled to the fiber tip. The output light emitted from the fiber tip has a divergent angle, which is changed with the surrounding refractive index. Using a low numerical aperture (NA) lens to collect the output intensity at a lower divergent angle, the measured image can be used to distinguish changes of the refractive index. We demonstrate the use of the camera of a smartphone to capture and analyze the image. The measured refractive index sensitivity for the axicon fiber tip has an intensity sensitivity up to 56,000%/RIU, and the detection limit is 1.79 × 10^−5^ RIU under an intensity stability of 1%. The intensity detection limits of the referenced fiber sensors range from 10^−5^ to 10^−6^ RIU [13,14]. The performance of the proposed axicon fiber is compatible with these works. However, as compared to previous works, the axicon fiber tip is easily combined with a smartphone device. It has the advantages of low cost, simple operation, and easy connection to the internet.

## 2. Theory

**Figure 1:**
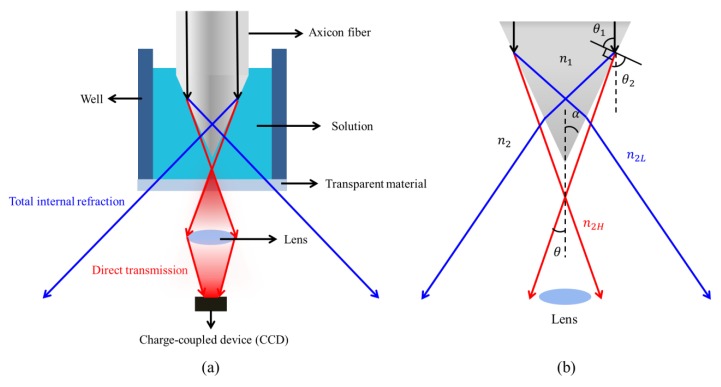
(**a**) A transparent axicon fiber tip combined with a camera device to detect the refractive index in the test solution; (**b**) the relation between the refractive index of the axicon tip and the divergence angle; and the divergence angle versus the refractive index at different angles of the tip.

Figure 1 shows a transparent axicon fiber tip and the optical path inside and outside the fiber tip. The fiber tip is immersed in an open well, which has a transparent bottom. After coupling light into a transparent axicon fiber tip, the guided wave is emitted from the tapered region into the test solution. The output light penetrates the transparent bottom and is collected by a lens at a small divergent angle. The collected optical signal is focused on a charge-coupled device (CCD) for analysis of the change of the optical intensity distribution.

According to Snell’s law, the difference in the refractive index between the axicon tip, $n_{1}$, and the surrounding environment, $n_{2}$, shown in Figure 1b, makes the angle of the input light change at the interface between the axicon tip and the surrounding environment. The angle of the refraction changes from $\theta_{1}$ to $\theta_{2}$. The emitted light, which is symmetrical along the center of the axicon tip, forms a divergence angle, θ, in front of the tip. The relation between the divergent angle and the incident angle is given by:

$${\theta =\alpha -(90-\theta }_{2}),$$

(1) $$n_{1}{\sin\theta }_{1}{=n}_{2}{\sin\theta }_{2}{=n}_{2}\sin\left(\theta -\alpha +90\right).$$

For the glass fiber, $n_{1}=1.46$ and $\theta_{1}=90-\alpha$. Based on the ray optics, when the cone angle, α, is smaller than 48.72°, the total internal reflection on the tip changes the emitting light (blue line) penetrating the other side of the axicon tip. The emitting angle from the tip is large so that the light cannot be collected by the lens. When the surrounding refractive index, $n_{2}$, is increased from the low value, $n_{2L}$, to the high value, $n_{2H}$, the internal total reflection disappears and the output light is emitted at a smaller divergent angle (red line), which can be collected by the lens.

**Figure 2:**
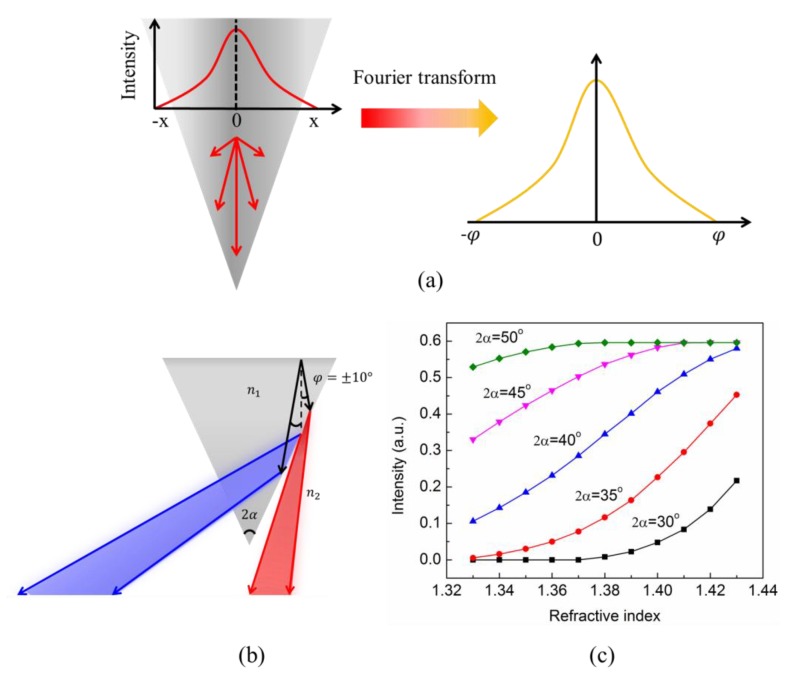
(**a**) Incident light in the fiber tip region can be transformed as different amplitude’s planar waves in various propagation directions; (**b**) the incident light propagates through the interface between the axion tip and the surrounding solution, plane waves (red part) can directly transmit to the solution at a lower divergence. Planar waves (blue part) with incident angles larger than the critical angle are emitted from the fiber tip at large divergent angles; and (**c**) the changes of the collected intensities (red part) for different axicon angles and various environmental refractive indices. These measurements were always performed with the same collection optics.

For the axicon fiber tip, the guided wave near the tip region has a very small spatial distribution. According to Fourier optics, the small light spot in the axicon tip can be decomposed by plane waves with different propagation directions as shown in Figure 2a. The incident light propagates not along the fiber but in a distribution known as the angular spectrum. When the incident light propagates through the interface between the axicon fiber tip and the surrounding solution, some plane waves (red part) can directly transmit to the solution at lower divergent angles as seen in Figure 2b. On the other hand, some planar waves (blue part) have incident angles larger than the critical angle and are emitted from the fiber tip at large divergent angles due to the total internal reflection. By using a lens with a small numerical aperture (NA) to collect the emitted light inside a small angle, the change of the optical intensity can be detected in various surrounding refractive index values. Figure 2c shows the calculated transmission intensity for different tip cone angles (2α) under various refractive index conditions. The incident angular spectrum has a Gaussian distribution with an angular range of ±10° in the tip. The refractive index of the surrounding medium ($n_{2}$) ranges from 1.33 (water) to 1.43. Figure 2c shows that cone angles of 35 to 40 degrees can have a large transmission intensity change for a different refractive index change. For a lower cone angle, most light is under total reflection. For a larger cone angle, the total internal reflection disappears and the transmission intensity change is small.

## 3. Fabrication

**Figure 3:**
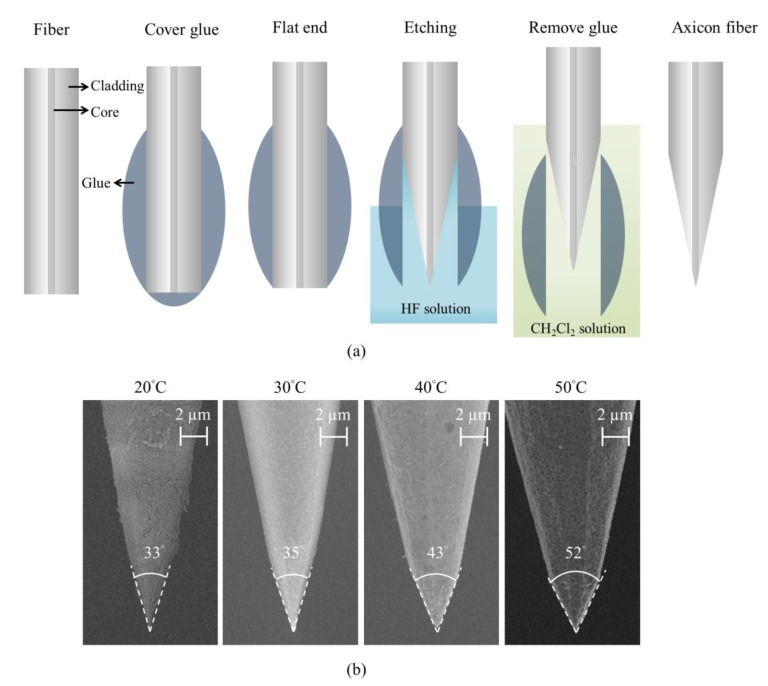
(**a**) Fabrication process from left to right; (**b**) the fabrication from a bare fiber to an axicon fiber with a different cone angle using a different etching temperature.

The axicon fiber tip was made by using an end-etching method for glass fiber [15]. A hydrofluoric (HF) solution was used to etch a single mode fiber (ThorLabs SM600) with a polymer protection coating to create an axicon shape in the end of the optical fiber. The fabrication details are as follows and are shown in Figure 3a: First, the fiber jacket was removed and a planar end surface was formed using a fiber cutter. The bare fiber was immersed in hot melting glue to provide a thick protective polymer layer. The polymer at the fiber end was further removed by placing the fiber end on a hot plate. The polymer-protected bare fiber was immersed in HF solution. Owing to the polymer protection, the fiber can only be etched from the tip end. The capillarity effect at the polymer and fiber interface resulted in an axicon shape at the end of the fiber. Then, the polymer-covered fiber tip was immersed in the CH_2_Cl_2_ solution to remove the polymer coating. From our previous study [16], the cone angle can be controlled by etching the fiber tip by the temperature. Figure 3b shows the SEM images of the fiber tips etched at different temperatures. The cone angle increased with the temperature. In our experiment, the cone angle ranges from 20 to 50 degrees. Between a 20 and 30 °C etching temperature, the cone angle falls in the optimal region as indicated in Figure 2c. Based on the simulation results, we used the axicon fibers made at room temperature.

## 4. Setup

**Figure 4:**
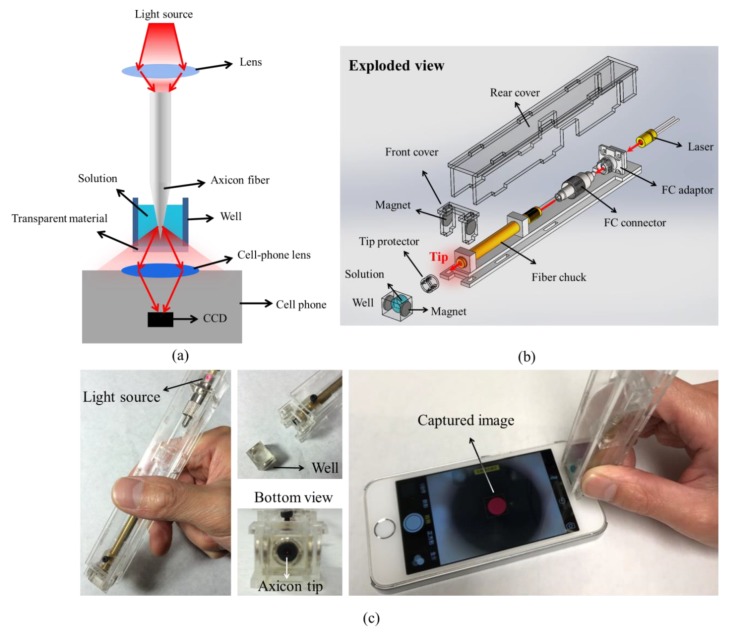
(**a**) The schematic of the optical axicon tip sensor combined with a smartphone camera for detection; and (**b**) the design and (**c**) photos of the portable axicon fiber tip sensor and detection using the front camera of a smartphone.

The axicon fiber tip combined with a smartphone for refractive index detection is shown in Figure 4a. The axicon fiber tip was immersed in a small-volume sample solution inside a well with a transparent bottom for transmission detection. By coupling the laser diode (650 nm wavelength) with the fiber, the light was guided in the fiber and emitted from the fiber tip. Different concentrations of the test solutions with various refractive indices changed the emitting optical intensity distribution. The camera of the smartphone, set at the same focal distance and same exposure time, was used to capture the output light intensity distribution. Figure 4b,c show the design of the device. The axicon fiber tip was combined into a portable sensor and measured by the front camera of the smartphone. The well covered with the test solution was fixed to the portable sensor using the magnet. The light spot from the axicon tip can be easily captured and displayed on the screen of the smartphone. Only a 200-μL solution was required for the measurement.

## 5. Results and Analysis

**Figure 5:**
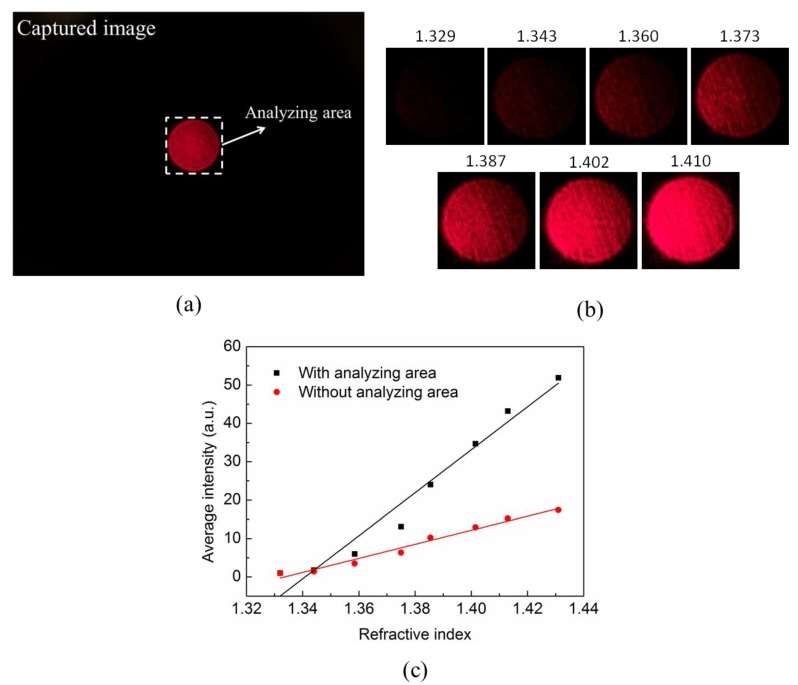
(**a**) The capture image from the front camera of a smartphone. The selected area for the intensity analysis; (**b**) captured image in the selecting area for test solutions with refractive indices; and (**c**) the relation between the average intensity and refractive index with and without the selective area.

Figure 5a shows the captured image and selective area for the intensity analysis. The captured image had a format of 24 bits Joint Photographic Experts Group (RGB JPEG). The center of the analyzing area is located at the center of the spot. The size of the analyzing area was manually selected to cover only the optical spot and remove the background area on the screen. Because the beam spot is surrounded by a black screen, there is a clear edge between the spot and the background. For automatic measurement applications, we can set a threshold value by turning off the laser. By selecting the area higher than the threshold value, the proper analyzing area should be defined automatically. We transferred the red pixels into integer values and took the average value of the selected area via a Matlab program. Figure 5b shows the selected image of the light spots that were emitted from the fiber tip in glycerol solutions with various refractive indices. Before the real sample measurement, a calibration curve for the tip sensor was established by measuring glycerol solutions with known refractive indices. The measured average intensity as a function of the refractive index is shown in Figure 5c. The intensity sensitivity of the sensor is defined as $\Delta I/\Delta I_{0}\times 1/\Delta n\times 100$%/RIU, where ΔI is the intensity change and $I_{0}$ is the reference intensity under the water condition. $\Delta I/\Delta I_{0}$ indicates the normalized intensity change. Δn is the refractive index change and RIU is the unit of the refractive index. The intensity sensitivity analyzed from the whole captured image was 18,291%/RIU. It is notable that the selective area plays an important role in enhancing the intensity sensitivity. For the whole image, although the image outside the red spot region is black, the CCD pixels still have some small values due to the background signals. It increased the $I_{0}$ and reduced the normalized intensity change. For the selected images, the average intensity slope was 560.12 per the refractive index. Therefore, the intensity sensitivity was ~56,000%/RIU. The change of the average intensity with the selective area analysis was enhanced three times due to the reduction of background signals. Based on the 1% intensity stability of the measurement system, the detection limit was 1.79 × 10^−5^ RIU.

**Figure 6:**
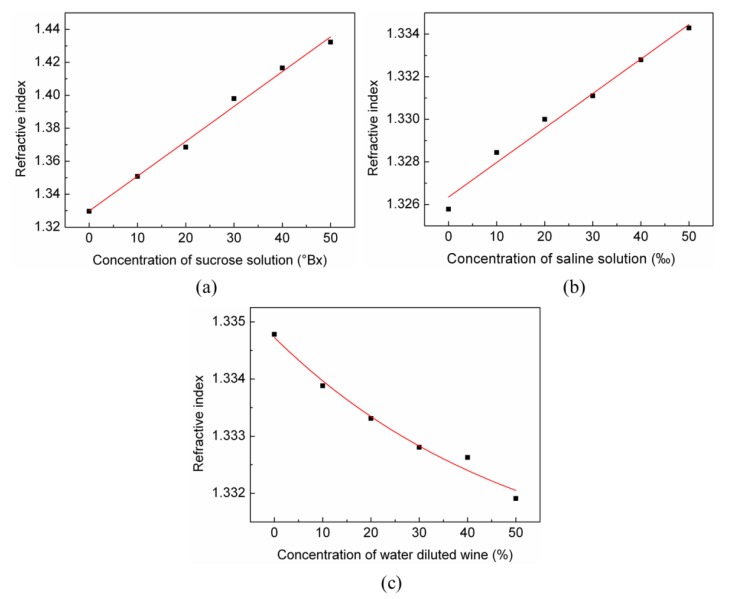
The measured refractive index for three different kinds of solutions. (**a**) Sucrose solutions with different degrees of Brix; (**b**) saline solutions with different permilles; and (**c**) water-diluted wines with different dilution rates.

By using the axicon fiber tip combined with a camera device, various solutions, such as sucrose solutions, saline solutions, water-diluted wine, etc, can be measured and identified. In the measurement, the measured average intensities were transferred to refractive indices using the calibration curve. The detection limits for different kinds of solutions were estimated by the refractive detection limit. The refractive indices of the sucrose solutions with 10, 20, 30, 40, and 50 °Bx (degrees Brix) are shown in Figure 6a, and the detection limit of sucrose solution was up to 8.86 × 10^−3^ °Bx. The refractive indices of saline solutions with 10‰, 20‰, 30‰, 40‰, and 50‰ are shown in Figure 6b. The detection limit of saline solution was 0.12‰. The refractive indices of water diluted wine with 10%, 20%, 30%, 40%, and 50% dilution rates are shown in Figure 6c, and the detection limit of the water dilution rate for wine was 0.35%.

## 6. Conclusions

We propose using an axicon fiber tip combined with a smartphone to make a portable, inexpensive, and high-sensitivity refractometer. The fiber tip was fabricated by the wet end-etching process at room temperature and formed a tapered angle of 35 to 40 degrees. The change of the light spot emitted from the axicon tip in different samples was captured and selected using the front camera of a smartphone. The captured images were transferred to refractive index values by using solutions with known refractive indexes. The result shows that the portable axicon fiber refractometer had a high sensitivity up to 56,000%/RIU, and the detection limit can reach 1.79 × 10^−5^ RIU. The detection device only needs a small sample volume due to the ultrasmall fiber tip’s area. The sensor can be used for different kinds of applications. We demonstrated the measurement for sweet, salty, and fake wines. For the sweetness measurement, sucrose with different degrees of Brix was tested. The detection limit was 8.86 × 10^−3^ °Bx. For the saltiness measurement, saline solutions with different concentrations were tested. The detection limit was 0.12‰. For the fake wine, we tested the water-diluted wine samples. The dilution could be identified and had a detection limit of 0.35%. The transparent axicon tip sensor combined with the camera device can also find applications in the food industry, environmental monitoring, and biomedical sensing.
